# Agro-Industrial Plant Proteins in Electrospun Materials for Biomedical Application

**DOI:** 10.3390/polym15122684

**Published:** 2023-06-14

**Authors:** Emilija Zdraveva, Višnja Gaurina Srček, Klara Kraljić, Dubravka Škevin, Igor Slivac, Marko Obranović

**Affiliations:** 1 Faculty of Textile Technology, University of Zagreb, Prilaz baruna Filipovića 28, 10000 Zagreb, Croatia; emilija.zdraveva@ttf.unizg.hr; 2 Faculty of Food Technology and Biotechnology, University of Zagreb, Pierottijeva 6, 10000 Zagreb, Croatia; visnja.gaurina.srcek@pbf.unizg.hr (V.G.S.); kkraljic@pbf.unizg.hr (K.K.); dskevin@pbf.unizg.hr (D.Š.); mobran@pbf.unizg.hr (M.O.)

**Keywords:** electrospinning, plant proteins, zein, soy protein, wheat gluten, biomedicine, wound dressing, drug delivery, tissue regeneration

## Abstract

Plant proteins are receiving a lot of attention due to their abundance in nature, customizable properties, biodegradability, biocompatibility, and bioactivity. As a result of global sustainability concerns, the availability of novel plant protein sources is rapidly growing, while the extensively studied ones are derived from byproducts of major agro-industrial crops. Owing to their beneficial properties, a significant effort is being made to investigate plant proteins’ application in biomedicine, such as making fibrous materials for wound healing, controlled drug release, and tissue regeneration. Electrospinning technology is a versatile platform for creating nanofibrous materials fabricated from biopolymers that can be modified and functionalized for various purposes. This review focuses on recent advancements and promising directions for further research of an electrospun plant protein-based system. The article highlights examples of zein, soy, and wheat proteins to illustrate their electrospinning feasibility and biomedical potential. Similar assessments with proteins from less-represented plant sources, such as canola, pea, taro, and amaranth, are also described.

## 1. Introduction

**Figure 1:**
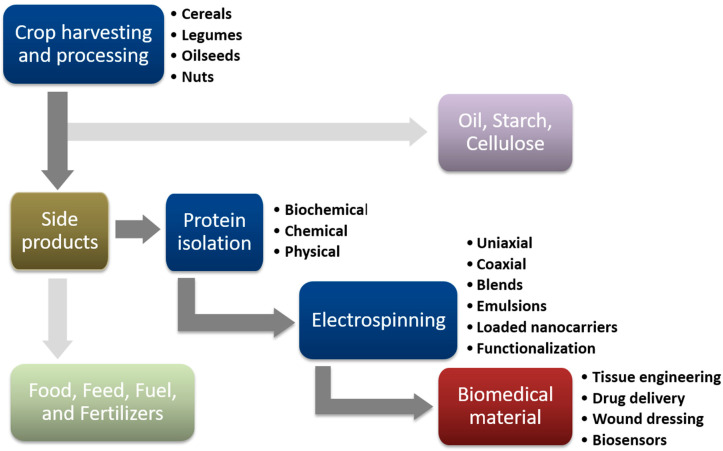
Agricultural plant proteins in production of electrospun biomedical materials. After crops are harvested and undergo primary processing to fabricate substances such as oil, starch, and cellulose, a significant quantity of vegetal side products is generated. Typically, they are diverted toward production of novel food, animal feed, fuel, or fertilizers. However, an alternative application involves utilizing the side products as a valuable source of proteins suitable for various electrospinning techniques. By turning the isolated proteins into fibers, materials with excellent bioactive and biomimetic properties can be created for diverse biomedical applications.

As a result of the fast population growth, it is expected that by 2050 the demand for food, feed, and fuel worldwide will grow by 70% (OECD/FAO, 2021). This increase in population, together with changing sociodemographic factors, pushes food manufacturers to provide highly nutritious food products with low impact on the environment. Due to rising interest in sustainable and eco-friendly diets, plant-derived proteins are becoming commercially more desirable [1]. Proteins of plant origin show advantages over animal proteins because they are naturally abundant and, as food product ingredients, beneficial for human health. Moreover, consuming vegetarian products reduces animal-to-human pathogen transmission and alleviates ethical concerns [2]. Apart from their dietary relevance, plant proteins are often used as components in many kinds of material, providing assets such as mechanical strength, water stability, fibrous texture, and most importantly, biocompatibility [3]. Proteins are biological macromolecules composed of amino acids with a very complex molecular structure. The amino acid side chain groups are responsible for their hydrophobicity and molecular conformation, which highly affects protein function. Many techniques have been developed to obtain proteins from vegetal sources including chemical: alkaline or saline extraction; biochemical: enzymatic digestion; and physical techniques relying on ultrasound, microwave, pulsed electric field, and high-voltage electrical discharge [4,5,6]. The choice of protein isolation and purification method depends greatly on the protein source, properties, and final purpose [7]. An environmentally sound strategy for minimizing waste disposal, utilizing resources to the fullest, and giving various products more market value, is the recovery of protein from agro-industrial byproducts (Figure 1). Oil cakes, byproducts of oil processing, are one of the most valuable sources for protein recovery since they are a high source of plant protein with a protein content of 15–50%. Their supply from major oil crops such as soybean, canola, and sunflower, or some alternatives rich in fibers, such as hemp and flax is quite considerable. Byproducts of cereal and tuber starch production, or legume processing are also good sources for protein recovery because of their availability and favorable amino acid content [5]. As natural polymers, plant proteins have been explored for biomedical applications for over a quarter of a century [8]. In comparison with animal-derived proteins, they show low immunogenicity and do not transmit animal-borne diseases. Their chemical stability, structural versatility, bioactivity, and high availability allow them to be engineered into different functional forms such as nanoparticles, nanogels, and nanofibers for various purposes [9].

The aim of this paper is to provide an overview of recent achievements in the biomedical application of plant protein-based materials fabricated through electrospinning. Electrospinning is a process used to produce nanofibers from a diversity of synthetic, semi-synthetic, and natural materials, including proteins. Plant protein-based electrospun nanofibers/matrices demonstrate tremendous potential in biomedicine since their fibrous structure mimics the extracellular matrix of animal tissues and as such facilitates cell immobilization and metabolite transfer. Moreover, these materials are easy to functionalize in order to modify their properties or entrap bioactive compounds that may achieve a certain effect upon their release. Plant proteins are often biocompatible and biodegradable, which is of extreme importance for their biomedical application. For example, electropsun maize protein, zein, applied on a tissue is decomposed by the body’s own physiological mechanisms that involve enzymes, pahgocytes, and local microbiota [10]. Some plant proteins even contain additional cell-recognition motifs that assist cells in distinguishing between one another, adhering to fibers, and migrating across the tissue [11]. The electrospun materials with potential biomedical purpose can be roughly classified as: materials for wound treatment; materials with capacity of controlled drug release and hence increased healing efficacy; materials that support cell growth and tissue regeneration; and materials that can be functionalized as biosensors for detection/quantification of certain microenvironental changes. However, electrospun plant protein fibers are predominantly investigated for making bioactive food packaging films or air filters, because it involves less rigorous sanitary regulation [12]. Yet, with continual progress in material science, biotechnology, and stem cell biology, plant protein nanofibers are receiving a lot of attention in the biomedical sphere. During the last twenty years, the number of papers about electrospun biomedical materials has been growing exponentially [13]. This is due to the increasing number of materials that has been tested, as well as the constant improvements in electrospinning techniques. With respect to synthetic, semi-synthetic, and other natural polymers, including proteins of animal origin, our focus here is solely on vegetal proteins sourced from agricultural waste.

There are not many reviews covering this particular topic, so we believe this is a timely contribution regarding the growing trends in development of biomedical materials and agricultural sustainability. Here we discuss proteins from widely available agricultural plants: maize, wheat, soy, canola, pea, potato, taro, and amaranth, that, according to FAO (Report 2020), make more than 30% of the global crop production. By presenting the findings of recent assessments with electrospun materials incorporating plant proteins, we highlight the advantages provided by these natural polymers. With attention given to the biomedical utilization, the technical aspect of the material production through electrospinning is slightly out of focus in this review. To our knowledge, the papers on similar topic are either less oriented to solely vegetal proteins, or they cover less recent studies [14,15]. The large majority of research we report here has been published within the past five years.

## 2. Electrospinning of Plant Protein Fibers—Modes and Purposes

**Figure 2:**
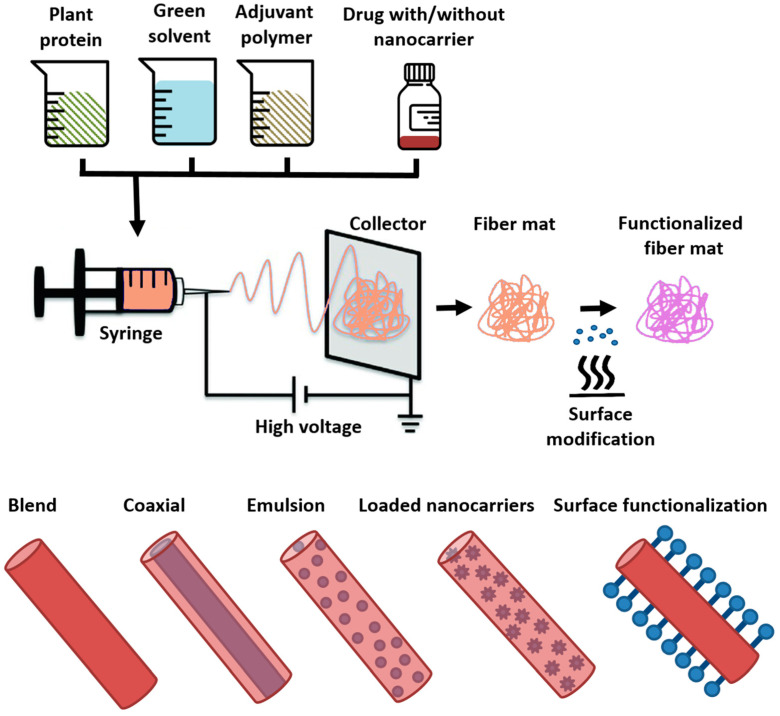
Production of different plant protein-based electrospun nanofibers. Plant protein-based electrospun fibers can have homogeneous cross-section obtained by blend electrospinning, core–shell configuration obtained by co-axial or emulsion electrospinning or a two-phase structure (with a discontinuous phase distribution) obtained by emulsion electrospinning; nano vehicles carrying small molecule drugs can be encapsulated into the plant protein electrospun fiber matrices; post-processing chemical treatments are used for the production of target functionalized electrospun fibers also; plant protein-based electrospun fibers used as drug delivery systems can be produced by all production procedures with the basic composition including the plant protein isolate, a non-toxic solvent, a co-polymer, and the drug compound with or without a nanocarrier.

Plant proteins are low in cost, have low immunogenic potential, and very much abundant natural polymers that have gained attention in recent years for the fabrication of electrospun nanofibrous materials and their application in advanced health care products (i.e., for wound healing, tissue engineering, etc.), food packaging, pharmaceutical industry, etc. [9]. Due to their complex structure, particularly secondary and tertiary, their spinnability may be difficult. Thus, in order to obtain continuous uniform fibers, the plant proteins require good solvent dissolution in a random coil conformation. The electrospinning technique requires electrostatic forces to stretch a viscoelastic polymer solution or melt for the formation of ultra-fine fibers with unique properties, such as: high surface to volume ratio, light-weight, adjustable diameter/morphology, and fibrous porous materials with high pores interconnectivity, as well as controlled functionality. Although simple in its popular device set-up the process is quite complex since it is based on both: the physical parameters phenomena, involving the high voltage power supply (electrostatic charges), flow rate, and needle to collector distance; as well as the rheological properties of the polymer solution, conductivity, viscosity, and surface tension. Having this in mind and the forth-mentioned protein structure, its spinnability should be properly designed by manipulating its conformation as well as aggregation property [16]. Other characteristics that affect the formation and uniformity of the protein electrospun fibers would be its molecular weight, surface charge, ionic, hydrogen and intra/intermolecular disulfide bonds, unfolding degree, and chain entanglement. The right solvent choice further supports proteins’ electrospinnability and affects final fiber properties [17,18,19]. The solvent providing high solubility causes denaturation of the plant protein or destruction of its bonds, thus the necessary chain pullability during electrospinning. There are also polymer carriers that can provide the protein with electrospinnability, thus the protein is blended with it and more easily electrospun into fibers. Such polymers usually include polyvinyl alcohol (PVA) and polyethylene oxide (PEO) [20]. These water-based polymers can maintain the protein function during electrospinning, but often proteins are combined with other synthetic polymers (polycaprolactone (PCL)) or their semi-synthetic equivalents (poly-L-lactic acid (PLA) and chitosan) that require harsh organic solvents. In the case of aqueous solutions, cross linkers are used in order to obtain water stability of the plant protein fibers, as well as their mechanical strength. The cross-linkers may also be natural or synthetic, and detrimentally toxic, hence they are rarely recommended. Apart from PVA and PEO, animal proteins (i.e., gelatin, whey protein and silk fibroin (SF)) can be used as carrier polymers for improving the spinnability of plant proteins. For example, whey protein isolate and soy protein isolate can form stable spinning solution with intermediate length gelatin [21]. Technologies reported for the production of plant protein-based electrospun fibers include: single nozzle blending, emulsion electrospinning, coaxial electrospinning, as well as electrospinning with the incorporation of nanocarriers and post-processing treatments (Figure 2), which is discussed in later paragraphs. Moreover, freshly fabricated fiber mats can be additionally functionalized and purpose adjusted, for example, by simple heating to attain hydrophobicity, or by grafting biomolecules such as peptides to achieve bioactivity [22]. Single nozzle blend electrospinning is conducted when two components are combined with the same dissolving solvent to form a homogenous solution. This is the most common technique of electrospinning as the plant protein is supported by another polymer compound which improves spinnability and usually provides the system a proper mechanical integrity. In emulsion electrospinning, the two compounds are separately dissolved in their respective solvents, while the two-phase solution is stabilized by an emulsifier under constant vigorous stirring. The as-prepared system can show fiber morphology with uniform polymer matrix (continuous phase) and spherical filler homogenously distributed along the fibers length, or a core–shell structure may be spontaneously achieved such as in coaxial electrospinning. To best of knowledge, these systems are reported to a lesser extent compared with the blend electrospun plant protein-based fibers. In coaxial electrospinning, two separate polymer solutions, usually immiscible, are electrospun simultaneously from two different containers through a coaxial nozzle to form core–shell nanofibers. This is a good way to encapsulate bioactive compounds in order to maintain their activity and control their release profile if necessary.

As earlier mentioned, there are two mechanism routes for the electrospinning of plant proteins. Firstly, a globular protein is dissolved in an ideal solvent, denatured in a random coil conformation, and spun as a single polymer. If the solvent is of low quality, the denaturation of the plant protein results in aggregation. Thus, the second route in this case provides the spinnability of the denatured protein with the help of the carrier polymer. The parameters that need to be monitored during electrospinning include the solution concentration that should be higher than the overlap concentration, while the viscosity should not be too high to ensure entanglement. The electrical voltage as well as the conductivity should also be optimal to further provide a workable condition for the process [21]. Generally, plant proteins are electrospun together with other well-spinnable polymers, except for zein or amaranth protein isolate, which are reported to be electrospun alone or with other polymers as well [16]. The most advantageous function of the plant protein fibers would be their ability to carry sensitive bioactive compounds (i.e., curcumin, quercetin, essential oils, etc.) and maintain their properties [23]. Specific drugs or bioactive substances can be loaded into these fibers based on their isoelectric property or the possibility to change their charge [24]. They are also biocompatible, biodegradable, and eco-friendly, and additionally, the electrospinning provides these materials with easily controlled porous interconnected structures, target fiber morphology, and functionality. The porosity of these electrospun materials is related to the fiber diameter, which can be controlled primarily by the solution concentration. Thus, higher solution concentration results in thicker fibers and greater pore sizes, which finally increases total porosity [25]. The morphology of the fibers can also be changed with the solution pH, for example ribbon-like smooth fibers are formed in acidic solution, while deformed beaded fibers are formed in alkaline solution [26]. The mechanical strength of the electrospun protein fibers can be altered, that is improved, by blending the plant protein with synthetic polymers, or with the addition of the cross-linking agents. Toxic cross-linking agents can also be replaced by electrospinning of multiple blend polymer systems together with the plant protein. In electrospinning, the biggest concern is the usual dissolution of the majority of polymers in organic highly toxic and carcinogenic solvents. Thus, to reduce possible hazards to human health and to maintain the activity of biological compounds, water-based dissolving polymers are generally suggested (or non-volatile solvents) for the formation of green nanofibers. On top of this, the stabilization of the same can also be conducted with the application of biobased cross-linkers. It was reported that green electrospun fibers compared with traditionally electrospun ones improve polymer crystal and ductile structure, promote cell growth with significantly greater cell number, and result in relatively the same cell matrix deposition, with glycosaminoglycan and collagen penetrating throughout the scaffold depths [27]. Generally, electrospun nanofibers are well-established in the field of pharmaceutical applications for the controlled delivery of drugs (antibiotics, antiseptics, and anti-inflammatory drugs) or biological compounds such as cytokines, hormones, and other signaling molecules. One of the ways to control the delivery of incorporated compounds in electrospun fibers is through the variety of the electrospinning technique, including: blend, emulsion, coaxial, triaxial, or multiaxial electrospinning, side-by-side electrospinning, drug-loaded nanocarriers (nanoparticles, nanotubes, microspheres, liposomes, etc.) electrospinning, as well as post-surface modifications [28,29]. Depending on the fabrication technique, the globular protein unfolds in the high quality solvent, while the active compound is: (1) added directly in the solution (blend electrospinning); (2) dissolved in the shell or the core solution together with additional polymer (coaxial electrospinning); (3) dissolved in the emulsion as the inner phase (emulsion electrospinning); (4) loaded in a separate nanocarrier and then within the plant protein nanofiber; or (5) added after electrospinning through surface functionalization [19]. The fabrication process is responsible for the fiber morphology or fibrous structure, which are both important in the design of the drug pace release mechanism. Another important factor is the electrospun fiber composition that includes both type of polymers used as well as drug properties (i.e., hydrophobic and hydrophilic) [28,29]. Depending on the final product’s therapeutic function, the release of the drug can be permediated in several modalities including: immediate or burst release (within several hours) [30], prolonged or sustained (it may take days to years) [31], and on-demand delivery or triggered by external stimuli (i.e., pH, temperature, light, magnetic field, etc.) [32] when necessary. The process of drug release includes the transportation of the molecule from the inner polymer structure to its outside surface and then to the release of the medium. The mechanisms that are involved in this process can be based on diffusion (through water-filled pores or the polymer matrix), hydrolysis, osmosis, erosion, swelling, polymer relaxation, degradation (polymer structure deformation or collapse), polymer–drug or drug–drug interactions [33].

## 3. Recent Trends in Biomedical Application of Electrospun Plant Proteins

### 3.1. Zein—Source and Properties

Zein represents the main group of storage proteins found in the seeds of maize. Commercial zein is essentially a byproduct of maize wet-milling in starch and oil technology. The maize protein content varies from 6% to 12% on a dry basis, of which zein accounts for 45–55%. Based on solubility, maize proteins primarily include albumin (soluble in water), globulin (soluble in a salt solution), glutelin (soluble in alkali), and zein (soluble in alcohol). The high amount of nonpolar amino acid residues and lack of charged amino acids are responsible for zein’s water insolubility. Biologically, zein is a mixture of proteins of various molecular sizes, solubility, and charge. According to solubility and sequence homology, it can be separated into three fractions labeled α-zein, β-zein, and γ-zein. The first one can be extracted using only aqueous alcohol, whereas the other zeins need a reducing agent in the solvent to be extracted. Commercial zein is mainly α-zein extracted by aqueous isopropyl alcohol, which selectively dissolves the protein and minimizes the other zein fractions [34]. Electrospinning of zein has many advantages; such as no need for aggressive solvents, adjuvant polymers, or cross-linkers. Its fiber-forming properties are based on the formation of hydrophobic forces, hydrogen bonds, and limited disulfide linkages between and within molecules. However, nanofibers fabricated from zein alone are brittle, so plasticizers are often incorporated to make electrospun matrices soft and permanently flexible. Due to its biodegradability and biocompatibility, zein has been vastly studied for use in nutrition and biomedicine, particularly for the fabrication of smart food, bioactive membranes, wound healing, controlled drug delivery, and 3D scaffolds for tissue regeneration (Table 1). For these purposes, zein can be combined with other biodegradable natural or synthetic polymers, in order to obtain desired properties and function. Several extensive review papers on the potential of zein-based materials for above-mentioned application were published recently [35,36,37,38].

#### Biomedical Application of Electrospun Zein

Zein nanofibers have become attractive to scientists developing biosensors for public health and environmental protection purposes. Based on the characteristics of electrospun matrix embedded components, biosensors with distinct functions are achievable. Highly enhanced sensitivity for the presence of carcinogenic acrylamide was obtained by fabricating zein mats decorated with the precise arrangement of gold and silver nanoparticles [39]. More recently, a biosensor based on electrodes coated with curcumin carbon dots and zein electrospun nanofibers, detected *S. aureus* in milk using electrical impedance spectroscopy [40]. Similarly, potential food poisoning c be prevented using a zein-based pH-responsive strip with alizarin loaded through intermolecular hydrogen bonding. The color of the biosensor showed visually recognizable changes in real-time for the different levels of the freshness of trout from a fish market [41].

The potential of zein as an active ingredient for controlled release and nanofiber-based delivery carriers has been investigated for over ten years [42]. Owing to its unique interfacial behavior, zein is especially suitable for the delivery of hydrophobic compounds [35]. Curcumin, a vegetal ingredient of exquisite antioxidant and anti-inflammatory properties, but of poor bioavailability is water-insoluble. It has been often tested for its bioactivity as part of materials for food packaging or inflammation treatment bandages [43]. In recent research, curcumin was entrapped in a zein/SF/chitosan polymer blend. Both mats, with and without loaded curcumin, exhibited excellent human gingival fibroblast adhesion and growth, suggesting feasibility for wound dressing [44]. Similarly, water-insoluble antioxidant, resveratrol, and silver nanoparticles were loaded to core–shell ultrathin fibers fabricated by coaxial electrospinning to ensure a synergistic antibacterial performance. Core fibers contained zein with silver nanoparticles, while shell was fabricated of PEO with resveratrol. The tests in vitro revealed bacterial pathogen growth inhibition, through sustained resveratrol release. Tested for improved packaging properties with cherry tomatoes, the electropsun material proved beneficial for keeping the vegetable quality [45].

Due to the demand for their prolonged therapeutic effect, antibiotics are another group of compounds tested in controlled release experiments, especially for wound healing and therapy. Using coaxial electrospinning, researchers produced mats composed of two-component fibers with zein as core and PCL as shell material; both polymers were with added antibiotic tetracycline. Besides improved antimicrobial activity, the matrices containing zein showed good water stability, ductility, and better fibroblast attachment compared with pristine PCL mats [46]. In another report, the same type of antibiotic was entrapped in graphene oxide nanoparticles that were loaded on zein-based electrospun fibers. The drug-loaded nanoparticles enhanced spinnability of the zein solution and physicochemical properties of the produced fibers. The composite showed a significantly prolonged drug release profile compared with the pristine zein nanofibers, as well as biocompatibility with fibroblasts in vitro [47]. The antibiotic-releasing bilayer membranes fabricated solely of zein were fabricated for the treatment of acute skin infections. Gentamycin-loaded zein fibers were prepared by electrospinning on the casted zein film surface. Mechanical properties of the bilayer membrane were found compatible with the skin tissue. In addition, membranes showed antimicrobial activity and supported the growth of keratinocytes and fibroblasts [48]. In a very recent study, thermally crosslinked zein/polyvinyl pyrrolidone electrospun membrane for propranolol hydrochloride transport through mucoadhesive delivery was designed and evaluated on ex-vivo porcine mucosal model [49].

Wound dressing strategies with polymer-based matrices standardly rely on keeping the functionality of bioactive ingredients loaded on the matrix. Famous for its skin-soothing properties, Aloe vera extract was loaded together with zinc-oxide nanoparticles on nanofibrous zein/PCL/collagen scaffolds. The composite had good mechanical properties and was found antimicrobial, and growth-supporting for fibroblast cells [50]. Skin damage recovery in vivo was evaluated in recent research. The induced skin burns in rats showed faster recovery without scars after treatment with verapamil-loaded zein/VPA electrospun mats [51]. Significant advancement in wound treatment techniques was achieved by increasing the flexibility and portability of the electrospinning equipment. The researchers carried out fabrication of zein/PEO membranes with clove essential oil fortification as a wound dressing technique on a live mouse model. The fabricated zein-based electrospun membrane presented excellent antibacterial properties and biocompatibility. Furthermore, the membrane was superhydrophilic in the simulated body fluid, which suggested the immense potential for absorption of wound exudate and preventing severe bleeding and infections [52].

Due to continuous research of other promising biopolymers, zein is being explored as a potential candidate to be used on tissue engineering applications. Glucose crosslinked gelatine/zein matrices were evaluated to regenerate bone in vivo and in vitro. The nanofiber scaffolds presented rapid mineralization in the mimicked body fluid containing bone minerals. The cytotoxic effect on MC3T3e1 cells of the nanofibrous scaffolds was negligible [53]. When cell growth factors are loaded on the bone-mimicking matrix, there is an improvement in the differentiation and promotion of the proliferation of osteoblasts. The recombinant human bone morphogenetic protein 2 and dexamethasone enhance tissue differentiation efficacy. Herein, it is necessary to maintain the stable release of the factor [54]. In a technically more complex approach, researchers designed a bilayer membrane with a unique drug delivery property combined with coaxial electrospinning and 3D printing aimed at periodontal tissue regeneration [55]. The honeycomb-like structure of PLA was fabricated by a 3D printer associated with the zein-based core–shell nanofibers to form the bi-layer membranes. The fabricated membranes exhibited appropriate wettability and mechanical strength for periodontal regeneration and higher stability against an aqueous environment, as well as a two-stage sustained release of tetracycline hydrochloride for eight days. Electrospinning may be a very suitable technique for making planar nanofibrous scaffolds for mimicking the skin cell environment. Electrospun zein/PCL/gum arabic nanocomposite scaffolds for skin regeneration were fabricated and fully characterized. Zein and gum arabic provide protein and polysaccharide content for skin regeneration and support, respectively, while PCL provides the mechanical elasticity and strength needed. Furthermore, it was found that the scaffold had porosity suitable for skin fibroblast proliferation and infiltration. Additionally, such composite scaffold demonstrated antibacterial properties due to the presence of cyanogenic glycosides in gum arabic, together with enhanced hydrophilicity [56].

**Table 1:** Electrospun zein and its potential biomedical application.

Protein	Adjuvant Polymer/Solvent	Electrospinning Type	Drug, Bioactive Compound and/or Condition Tested	Biomedical Effects and Suggested Application of the Electrospun Material	Reference
Zein	None/Acetic acid	Uniaxial	Curcumin carbon dots	Biosensors for bacterial contamination	[40]
	None/Ethanol	Uniaxial	Alizarin	Biosensors for bacterial contamination	[41]
	SF, Chitosan (Ch)/Formic acid	Uniaxial	Curcumin; Zein with and *w*/*o* adjuvant polymers	Better fibroblast growth in blends with SF and Ch; Sustained drug release; Wound dressing	[44]
	* C: PEO (no Zein)/Ethanol ** S: None,/Ethanol	Coaxial	Resveratrol, Nanosilver; Monolyth PEO and Zein vs. Core–shell PEO-Zein	Improved drug release with C-S system, Pathogen growth inhibition; Food packaging	[45]
	* C: None/Ethanol ** S: PCL, PEO (no zein)/Acetic acid	Coaxial	Tetracycline; Monolyth PCL vs. two Core–shell systems: Zein-PCL, Zein-PCL/PEO	Improved drug release with Zein-PCL, Better fibroblast adhesion comparatively to pristine PCL fibers; Wound dressing.	[46]
	None/Dimethylformamide	Uniaxial	Tetracycline loaded on graphene oxide particles (GO);	Improved drug release with GO; Wound dressing.	[47]
	PCL, Collagen/Chloroform, Ethanol	Uniaxial	Aloe vera, ZnO; Various Zein/PCL ratios	PCL improves blend performance, Antibacterial, Fibroblast adhesion, Sustained drug release; Wound dressing, Skin regeneration	[50]
	PEO/Ethanol	Uniaxial	Clove essential oil	Antibacterial in vitro; Wound dressing in situ on a mice model	[52]
	PVA/Ethanol	Uniaxial	Verapamil; PVA/Zein vs. PVA/Alginate	Sustained drug release better in PVA/Zein blend; Burn wound healing in vivo better with PVA/Alginate blend	[51]
	Polyvinyl-pyrrolidone/Ethanol	Uniaxial	Propranolol	Favorable drug release and cytotxicity in vitro, and mucosal drug delivery ex vivo	[49]
	None/Ethanol	Uniaxial on zein film	Gentamycin; Mulitlayer membrane system	Sustained drug release; Skin regeneration	[48]
	PCL, gum arabic (GA)/Acetic acid, Formic acid	Uniaxial	Various polymer ratios in Zein/PCL/GA blends	Presence of GA improves fibroblast adhesion and antibacterial properties; Skin regeneration	[56]
	* C: PEO (no Zein)/ Hexafluoroisopropanol ** S: PCL/Hexafluoroisopropanol	Coaxial on 3D zein/PLA platform	Curcumin, Tetracycline, β-glycerolphosphate	Sustained drug release; Periodontal tissue regeneration	[55]
	Gelatin/Acetic acid	Uniaxial	Glucose-crosslinked Zein/Gelatin blends as bone tissue scaffolds	Cranial bone regeneration in vivo improved with crosslinked polymers	[53]
	* C: PLA (no Zein)/Hexafluoroisopropanol ** S: None/Hexafluoroisopropanol	Coaxial	rhBMP2, Dexamethasone	Sustained drug release, Mesenchymal stem cell growth; Bone regeneration	[54]

* C—Core; ** S—Shell.

### 3.2. Soy Protein—Source and Properties

Soy protein (SP) is an abundantly available and cheap plant protein isolated from soybean after applying oil extraction. It consists of a mixed assortment of proteins with molecular weights ranging from about 140 to 300 kDa and with different physicochemical properties. The majority of soy proteins are storage proteins, 90% of which are globulins represented by several types of water-extractable fractions that differ in size and quantity [57]. SP contains polar, non-polar, and charged amino acid residues, including tyrosine, lysine, phenylalanine, leucine, glutamate, and aspartic acid that can interact with various biologically active molecules and drugs, the most common interactions being hydrogen bonds, hydrophobic interactions, and van der Waals forces. In addition, SP has good biocompatibility, has been extensively used to prepare nanoparticles, hydrogels, and emulsions, and has broad application prospects in the functional food and biomedical fields [58]. Compared with zein and wheat gluten, SP is not easy to electrospin on its own. Polymers such as PVA [59], PCL [60], PEO [61], PLA [62], or some other proteins are often added to enhance biomedical usability of the electropsun SP by ensuring smooth fiber morphology and elasticity of the material (Table 2).

#### Biomedical Application of Electrospun Soy Protein

With a desire to minimize involvement of potentially toxic organic solvents, sustainable strategies for waterborne electrospinning of SP/PEO blend were proposed recently and proved fully biocompatible in vitro [63]. A food-grade component mix of SP, whey protein, and a starch hydrolysate maltodextrin, showed better spinnability and hence superior fiber quality. This improvement was the result of protein glycation with reducing polysaccharides because of the close contact of the reactants inside the fibers achieved by the stretching and bending motions of the jet during electrospinning [64]. Loading SP matrices with food-grade antioxidants and antiseptics might be suitable for food or biological material storage. In that manner, ultrafine fibers carrying ginger essential oil were produced using a polymeric blend of SP, zein, and PEO. This material tested growth-inhibiting for five bacterial species [65]. Very recently, matrices of SP/PVA nanofibers containing different ratios of essential oils from two culinary plants, showed growth inhibition of several Gram-positive and Gram-negative pathogens [66]. Over 10 years ago, it was reported that electrospun SP blended with PEO exhibited inherent antibacterial qualities against *E. coli* and *S. aureus* [61]. This may be due to the presence of residual isoflavones, which have demonstrated antioxidant, anti-inflammatory, and cytostatic properties, but are also known to have an adverse effect on nitric oxide production, resulting in delayed wound healing [67]. Moreover, SP isolates contain two major allergens, glycinin and conglycinin, which might counteract the healing process [68]. Despite these facts, several researchers have focused on the use of soy-based nanofibers for wound dressing and drug release purposes testing both in vitro and in vivo models. Enhanced antibacterial activity was obtained by fabricating uniform nanofibers based on alginate, PEO, and SP blended aqueous solutions with vancomycin antibacterial drug loading. The fibers provided antibacterial activity against *S. aureus* related to the released vancomycin dose, while the fiber assessment in vitro with human dermal fibroblasts confirmed non-cytotoxicity and biocompatibility [69]. The same team carried out coaxial electrospinning with SP, PEO, and alginate blend containing tetracycline in core, and PCL in shell. It took the antibiotic several times longer to be released from the core–shell fabricated system than from the monolithic fibers, i.e., without the shell [70].

Confirming biocompatibility of electrospun fibers using tissue culture is a crucial step in evaluation of their biomedical relevance. SP/PCL blend containing tea tree oil showed antioxidant, antibacterial, and mouse fibroblast growth-supporting properties [71]. Electrospun mats fabricated of SP and PVA fibers with analgesic drug ketoprofen-preloaded tubular mineral sepiolite, demonstrated increased mechanical strength, and a much better drug release profile [72]. In a related manner, the composite mats were fabricated using SP/hydroxyethyl cellulose/PVA loaded with halloysite nanotubes capturing the anti-inflammatory drug diclofenac sodium. The presence of mineral nanotubes ensured sustained drug release from the mats for over two weeks. The composite showed antibacterial properties as well as cell biocompatibility in vitro [73].

Of all the plant proteins described here, SP is the most convenient substrate for cell adhesion owing to its amino acid composition. This clearly reflects in its increased assessments as tissue engineering and cultivated meat cell scaffold [74,75]. Because the porous nanofibrous matrix makes cells attach easier and nutrients more readily available, electrospun SP/PVA blend has been demonstrated to better support cell proliferation in vitro than solvent-cast film [11]. Some peptides in SP have amino acid sequence that serve as the anchorage site for receptor-based cell attachment. In that sense, human mesenchymal stem cell studies showed successful cell adhesion and proliferation on the SP/PEO fibers with optimized morphology and produced from an alkaline blend solution [76]. Electrospun materials fabricated only of SP fibers have also been investigated. In that case, SP isolate has been dissolved in an aqueous solvent system containing reductant. The obtained solution was electrospun into bulky, water-stable scaffolds composed of ultrafine fibers. An in vitro study showed that the 3D soy protein scaffolds well-supported uniform distribution and adipogenic differentiation of adipose-derived mesenchymal stem cells [77]. More recently, SP and silk fibroin dissolved in formic acid were used to fabricate electrospun mats that supported the growth of skin fibroblasts but reduced the proliferation of melanoma cells. Furthermore, the nanofibrous scaffold demonstrated its suitability for skin wound dressing in vivo [78]. In another study, SP fibers provided more favorable conditions for the maturation of induced retinal pigment epithelium (iRPE) sheets in vitro, than synthetic PCL-based matrices. It is worth pointing out that SP matrices were produced through a method known as blow electrospinning where air pressure facilitated fiber dispersal from SP/PEO water solution. The comparative transcriptome analysis in conjunction with principal component analysis demonstrated that iRPE on nanofibrous scaffolds, either natural or synthetic, matured more consistently than on nonfibrous substrates [79]. After optimizing process parameters, a group of researchers produced a novel SPI/PLA highly oriented nanofibrous conduit for peripheral nerve defect regeneration in the rat model. The material had improved tensile strength, surface wettability, and in vivo degradability, directing in vitro neural cell growth and extension, enhancing neurite outgrowth, and promoting in vivo nerve regeneration [80].

**Table 2:** Electrospun soy protein and its potential biomedical application.

Protein	Adjuvant Polymer/Solvent	Electrospinning Type	Drug, Bioactive Compound and/or Condition Tested	Biomedical Effects and Suggested Application of the Electrospun Material	Reference
Soy protein	Zein (Z), PEO/Acetic acid, Ethanol	Uniaxial	Ginger essential oil in SP/Z/PEO blend	Antibacterial; Food packaging	[65]
	PVA/Acetic acid	Uniaxial	Plant essential oils in SP/Z/PEO blend	Antibacterial; Food packaging	[66]
	PCL/Acetic acid	Uniaxial	Tea tree oil in SP/PCL blend	Antibacterial and Fibroblast scratch test in vitro; Food packaging, Wound dressing	[71]
	PEO, Alginate (A)/Alkaline solution	Uniaxial	Vancomycin, SP/PEO/A blends vs. PEO/A blend	Improved drug release from blends with SP	[69]
	PVA/Alkaline solution	Uniaxial	Ketoprofen loaded on sepiolite, in SP/PVA blend	Improved drug release with nanocarriers	[72]
	Hydroxyethyl cellulose (HEC)/Water	Uniaxial	Diclofenac sodium loaded on halloysite nanotubes in SP/HEC blends	Improved wound dressing and drug release with nanocarriers	[73]
	* C: PEO, Alginate (A)/Alkaline solution ** S: PCL (no SP)/Dichloromethane, Dimethylformamide	Coaxial	Tetracycline; Monolyth SP/A/PEO vs. Core–shell system SP/A/PEO -PCL	Drug release improved with coaxial system; Biocompatibility in vitro; Wound dressing	[70]
	PEO/Alkaline solution	Uniaxial	Optimization of SP/PEO fiber morphology	Mesechymal stem cell proliferation; Tissue regeneration	[76]
	None/Buffer solution, SDS, Cysteine	Uniaxial	2D vs. 3D cell scaffolds	Stability of 3D scaffolds, Mesechymal stem cells proliferation; Tissue regeneration	[77]
	SF/Formic acid	Uniaxial	SP/SF blends vs. pristine SP and SF, and casted SF discs	Better skin tissue regeneration in vitro and wound healing in vivo	[78]
	PEO/Water	Uniaxial, blow and standard electrospinning	SP/PEO fibers (blow electrosp.) vs. PCL fibers (stand. electrosp.)	Equal or better cell growth on SP/PEO; Retinal epithelium regeneration	[79]
	PLA/Hexafluoroisopropanol	Uniaxial, highly oriented	SP/PLA blends vs. pristine PLA; Different collectors	Superior peripheral nerves regeneration with SP/PLA conduits	[80]

* C—Core; ** S—Shell.

### 3.3. Wheat Gluten—Source and Properties

Wheat gluten (WG) is obtained after wheat dough is washed to remove water-soluble components. The dry leftover substance contains 75–85% protein and the rest are carbohydrates and lipids. Gluten is unique in terms of the amino acid composition and contains high amounts of glutamic acid, proline, and low amounts of amino acids with charged side groups, which makes it water-insoluble. The composition of WG is complex and includes glutenin and gliadin proteins. Glutenin is polymeric protein composed of subunits linked together through disulfide bonds; these subunits are classified into two groups: high-molecular weight and low-molecular weight glutenin subunits. Gliadins are monomeric proteins that are classified, in order of decreasing mobility in acidic polyacrylamide gel electrophoresis, as α-, β-, γ-, or ω-gliadins. The solubility of these proteins is determined by disulfide bonds. Gliadins are soluble in alcohols, while glutenins are soluble in acidic solvents. Although dietary intake of gluten is associated with metabolic disorders among celiac disease populations, there is no report to date about allergy related with electrospun WG-based coatings or films [81].

#### Biomedical Application of Electrospun Wheat Gluten

It is believed that the gluten fiber formation by electrospinning is a result of both chain entanglements and the presence of reversible junctions in the protein, in particular, the breaking and re-forming of disulfide bonds that occur via a thiol/disulfide interchange reaction. However, heat and high pressure imposed on gluten induce conformational changes that may prevent fiber formation [82]. One study suggests that very thin porous membranes composed of oriented WG fibers can be set up as a thermally stable and prolonged-release system of urea [83]. The hydrogen bonds between urea and WG proteins were fabricated by soaking the electrospun membranes in a urea solution. The urea release rate was then measured while having the urea-loaded membranes immersed in water. Since urea is known as a keratolytic, similar systems might be developed to help treat skin conditions and wound healing. WG can also be used to increase the protein content in fibrous blends with other plant-derived and protein rich substances such as soy flour and/or polyvinyl alcohol (PVA). The fiber cross-linking can be performed with glutaraldehyde [84] or preferably with some green alternative such as oxidized sucrose [85]. The blends were applicable as nontoxic and biodegradable adsorbents for extraction of nanoparticles from water. Yet in another study, WG solubility in aqueous solution was increased by using PVA that was partly thiolated with DTT. This ensured the beneficial combination of properties by keeping the polymer ability to make hydrogen bonds with WG, as well as by enabling the polymer to reduce disulfide bonds in the protein molecule [86]. Using non-reducing conditions and sonication improved WG solubility in SDS-phosphate buffer. Moreover, post-heat treatment of electrospun protein fibers played a significant role in inducing a higher degree of protein cross-linking while creating a cotton-like structure with blood absorption capacity and water stability appropriate for medical application [87]. Increased fiber quality, as well as functionalization capacity of gliadin electrospun matrices was achieved with the addition of polysilsesquioxane. This silicio-organic compound reduced standard viscosity of the protein solution and ensured obtaining tinner fibers [88]. Another group of researches incorporated glycerol monolaurate into wheat gluten-based film, and found that the addition improved the film’s water stability and antimicrobial activity against *S. aureus* and *E. coli* [89]. The same team produced a WG/zein blend that was glycated with xylose through the Maillard reaction. The blend had a higher thermal stability, water vapor resistance, water stability, and elasticity than the non-glycated one [90].

Being spinnable on its own or with other hydrophilic polymers makes WG-based mats an attractive system for studying controlled drug release in therapy. The kinetics of azathioprine release improved when electrospun WG fibers included PVA, which ensured thicker fibers and better material stability. Experiments in vitro showed that, while the WG component was degrading, the drug was released more gradually thanks to its interaction with PVA. The WG/PVA mats also proved non-toxic for endothelial cells [91]. An interesting approach in drug release control was tested using coaxial electrospinning of gliadin. The ultra-thin shell of the fibers was gliadin-based, while the encapsulated core was ketoprofen-gliadin nano-composite. In comparison with unshielded fibers, the coating layer ensured a better sustained release of the ketoprofen over a period of 16 h with almost complete content release and no initial burst effect [92]. A brief overview of WG utilization in biomedicine is shown in Table 3.

### 3.4. Other Plant Protein Sources

Here we discuss the progress made in creating electrospun materials that incorporate proteins derived from agro-industrial plants that have received limited attention or have been only recently reported in this field. Over the past several years, there has been a substantial increase in the assortment of plant-derived proteins used in electrospinning, resulting in varying degrees of bioactivity and biomedical functionality in the resulting nanofibrous matrices.

#### 3.4.1. Legumes

The soybean, being a prominent example of leguminous plants in the field of protein electrospinning, has spurred the investigation of proteins from other legume species as well. Using pea proteins (PPs) became attractive because pea seeds have lower levels of proteinase inhibitors and phytic acid, which reduces the risk of allergic reactions in humans compared with soybean. Similar to SP, PP can be used to impart functional properties such as emulsification, gelling, and water-holding capacity in foods and bio-materials [93]. The components of PP are globulin (65%) and albumin (25%) with a small amount of water-insoluble prolamin (5%) and glutelin (5%) [94]. While globulin is the major storage protein and soluble in salt solutions, albumin is soluble in water and regarded as a metabolic and enzymatic protein with cytosolic functions. However, electrospinning PP is difficult due to its globular structure and the lack of molecular entanglement [95,96]. PP isolate was electrospun with carbohydrate polymer pullulan by using a green electrospinning technique. Analysis of produced nanofibers indicated that the protein and the polysaccharide were in an entangled form. Thermal crosslinking of the nanofibers was also examined and showed that the thermic treatment of electrospun mats provides preferable hydrophobicity compared with the non-crosslinked samples [96]. Technofuncionality, including solubility, of PP was improved by heat-controlled glycation with maltodextrin, before needleless electrospinning [97]. Fibers produced using various solvents or PVA have shown varying sizes and artifacts. Despite this, studies have shown that nanofibers fabricated of pea protein can effectively combat *E. coli* and *L. monocytogenes* when combined with antibacterial agents such as cinnamaldehyde [95]. Cinnamaldehyde also has anti-inflammatory and other antimicrobial, antifungal, and anti-biofilm properties, making it a potential candidate for wound healing [98]. Interestingly, artificial intelligence has been utilized to discover an anti-aging peptide in the pea protein genome that can be used for wound healing. This peptide has been found to enhance the proliferation and production of keratinocytes and fibroblasts, resulting in a 40% reduction in wound area in vitro after 48 h compared with the control. Therefore, its use in wound healing should be considered [99]. The first report on common bean protein (BP) electrospinning was provided recently [17]. BP is characterized by its well-balanced amino acid content, rich in essential amino acids such as lysine, tyrosine, and phenylalanine. Phaseolin is the major storage protein in bean seeds and represents about 30–50% of the total protein content. It is a glycosylated protein with a trimer quaternary structure. Lectin proteins and protease, trypsin, and Bowman–Birk inhibitors are other important proteins found in beans [100]. The functional properties of bean protein such as solubility and emulsifying capacity from flours and BP isolates have already been evaluated [101]. According to the study [17], the denaturation of the protein isolates was not sufficient to form fibers. The fiber-like morphologies were only obtained when BP was dissolved in hexafluoroisopropanol, but traces of this toxic solvent were found in electrospun matrices. In this regard, using formic acid solutions would be more convenient, improved with the addition of a surfactant. Protein isolate of another legume was assessed in electrospinning experiments for the first time last year [102]. Typically, the isolate is a byproduct of peanut flour production. Using PLA as an adjuvant polymer, the research showed that by increasing the protein portion in the electrospun blend, the tensile strength of the nanofibers decreases while their hydrophobicity increases.

#### 3.4.2. Oil Crops

Protein isolates from oilseed crops, such as canola or sunflower, refer to the group of proteins that are recovered from the byproducts generated during the oil extraction process. Since the protein content is relatively high (20–35%), it is reasonable to explore its utilization in more inventive way, such as electrospun materials. The major storage proteins in canola or rapeseed are cruciferin, a large heterodimer globulin and napin, a smaller al-bumin, which together comprise 85–90% of the total proteins. Moreover, present in small amounts are structural proteins and metabolic proteins [103]. Alkali-extracted and acid-precipitated canola proteins (CPs) have low solubility around the pH that is employed in protein recovery, e.g., pH 3, 4, or 5 depending on the process. Napin-rich protein shows solubility values >90% in the range of pH 2 to 10, which is a unique characteristic for a plant (seed) protein. Conversely, cruciferin-rich protein isolates show lower solubility in the same pH range [104]. In a recent study of CP nanofiber formation, the protein isolate underwent pH shift with or without ultrasound assistance, prior to electrospinning the protein solution enriched with PEO [105]. The study indicated the effectiveness of ultra-sonic treatment during alkaline pH shift on the physicochemical properties of the CP solution (solubility, viscosity, and conductivity) as well as the structural refinement of the produced fibers. Furthermore, the antibacterial activity of electrospun matrices loaded with clove essential oil was assessed. This oil, besides being antiseptic, is famous for its analgesic and anesthetic properties [106]. Therefore, such modification of CP-based materials can be applied for the production of pain-reliving pads or food packaging material.

Concerning its unattractiveness as a food supplement, mostly due to its undesirable dark color, sunflower protein (SFP) isolate has also been evaluated, as a component of electrospun films. Much like other oilseeds, sunflower seeds contain around 20% of proteins that are 70% oligomeric globulin and 25% water-soluble albumin. In one paper, different ratios of the isolate were combined with PVA in an attempt to prepare a solution feasible for electrospinning. The authors used FTIR analysis to investigate the protein denaturation and interactions between SFP and PVA after heat treatment-induced hydrophobicity [107].

#### 3.4.3. Tubers

The proteins of starchy root vegetables such as common potato, and taro, were recently tested for fiber formation and functional matrix design by electrospinning. The protein sources are tuberous roots or underground stems of the plants. Potato cultivation is spread worldwide while taro, a native to southeast Asia, is specific to the tropics. In comparison with other food crops, protein production of potato per hectare and per day is second after wheat [108]. Fresh tubers are rich in starch and have a variable amount of protein (1–5%). The majority of the soluble protein fraction are albumins (55%) and globulin (25%) [109]. They often obtain electrospinning experts from the residual streams of starch production. In order to enhance polymer physicochemical stability, taro proteins (TPs) were crosslinked with chitosan and PEO using either a heat treatment or glutaraldehyde vapor. The in vitro antibacterial activity of the crosslinked nanofibers showed a stronger bacteriostatic effect on *S. aureus* than on *E. coli.* Moreover, the morphology and proliferation of human skin fibroblast seeded on the electrospun matrices becomes improved with the introduction of higher TP content in the polymer blend. These results suggest the feasibility of crosslinked electrospun TP–chitosan–PEO nanofibers for bioactive wound dressing materials [110]. With less focus on the biomedical aspect, two studies were performed about fibers of potato protein (PtP) and maltodextrin obtained by needleless electrospinning [111,112]. The earlier of the two describes the fiber properties after varying PtP and maltodextrin content and heat-inducing glycation process between the protein and polysaccharide. In the later study, the activity of trypsin inhibitor (a minor albumin fraction) within the electrospun fibers was investigated. It showed that the higher PtP content in the blend provides higher inhibition activity. Furthermore, knowing that trypsin cleaves next to lysine, the authors could not find a clear correlation between the number of free lysine groups in the fibers and the inhibitory effects on trypsin. Glycation of the two polymers affects the functionality of the electrospun matrices in a yet indefinite manner.

#### 3.4.4. Amaranth

Amaranth is a pseudocereal with a relatively short life cycle that requires little water, and it is capable of growing at a wide range of altitudes. These advantages, including its low cost, are responsible for its current growing use and application in food production. The amaranth seed has a high protein content (14%) compared with many common cereals. The protein fractions of amaranth are primarily composed of albumin (65%) and globulin (17%), while prolamin and glutelin are present in lesser amounts. Amarantin (a fraction of globulin), prolamin, and glutelin have poor solubility in water which makes their processing and hence the application difficult [113]. Experimenting with electrospun amaranth protein (AP) started more than 10 years ago [114]. The researchers blended AP with carbohydrate pullulan using formic acid as a solvent. Good fiber structure in blends with greater protein contents was only obtained upon surfactant addition. In its following publications, the research team loaded curcumin [115], as well as ferulic acid and quercetin [116] in the fiber matrix and observed that the antioxidant activity of the entrapped compounds was maintained after an in vitro digestion of the matrix. A higher proportion of AP in the polymer blends affected the compound diffusion, thus resulting in a more controlled release. Besides having antioxidant properties, the tested compounds are known for their anti-inflammatory or antiallergenic properties which altogether identify the biomedical functionality of the produced electrospun materials. Moreover, in another study using similar electrospinning conditions, the loading of nisin in matrices was evaluated. Nisin is a heat-stable antibacterial peptide often used as a food preservative. The result of the study showed the bacteriostatic activity of nisin against a food-borne pathogen [117]. Since that last report, to our knowledge, there have been no newer publications about using AP in electrospun materials.

A concise summary of biomedical utilization of electrospun pea, canola, taro, and amaranth proteins is presented in Table 4.

## 4. Conclusions, Prospects and Challenges

**Figure 3:**
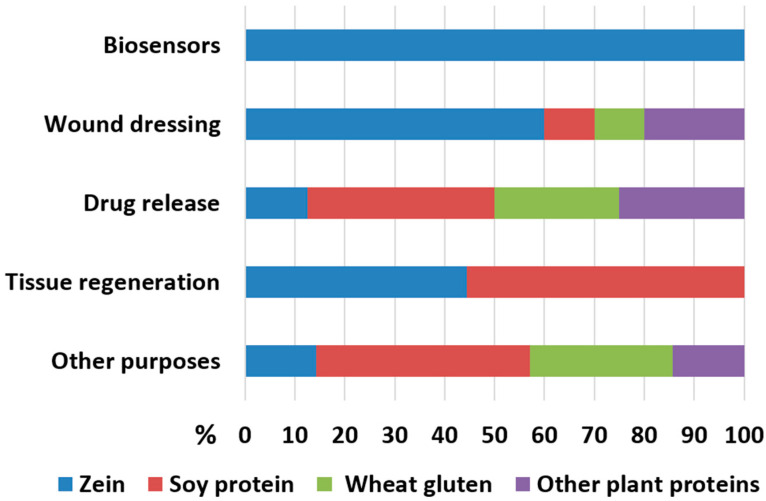
Distribution of electrospun plant proteins in proposed biomedical applications. The percentage values in the chart are based on the studies mentioned in this review. Apart from the studies on amaranth, the large majority were published during the past five years.

Driven by demand for the alternative to animal proteins, byproducts of agro-industrial crop processing are an easily accessible protein source with enormous economic potential. Plant protein-based materials produced through electrospinning are raising attention due to their bioactive properties that make them applicable as fibrous matrices for treatment of damaged tissue, sometimes functionalized with drugs, or entangled with viable cells. When the contact with living tissue is not the endpoint, the protein fibers are used for making food-borne pathogen sensors, antibacterial wrapping, liquid absorbents, or air filters (Figure 3). However, the physicochemical diversity of the proteins, as well as the technicality of electrospun fiber production, makes these applications variably successful and restricted. From the growing number of studies that suggest some kind of biomedical utilization of electropsun plant proteins, one can immediately notice the great versatility of zein. This should not come as a surprise since zein is an amphiphilic protein, easy to functionalize, and often used in formulations that require resistance to water, heat, and abrasion [36]. The second most widely used fibers, with the relatively highest efficiency in cell growth support, are fabricated of SP. This protein exhibits a variety of peptides that promote migration and cell proliferation, key factors for tissue regeneration, among them is lunasin with RGD-like sequences that promote stable cell adhesion [75]. Owing to its numerous hydrogen bonds, WG is quite soluble in aqueous systems and hence very reliable in hydrophilic physiological environments [118]. In such conditions, WG fibers can undergo gradual decomposition for sustained drug release or a steady process of wound healing. Other proteins, although less available, need obviously more attention of experts in order to become relevant in a particular biomedical niche. Improving nano-structure of protein fibers and their systematic assessment in blends or composite matrices together with better control over the active compound binding and release, or improved cell adhesion, can positively impact their fate.

Although agricultural plant-based waste offers a plethora of protein sources for the production of electrospun materials, several ongoing limitations make it necessary to resolve the challenges for their biomedical applications. The major concern is the purity of proteins isolated from agricultural waste, which can highly restrict their clinical use. Impurities, such as remaining compounds from plant tissue, or residual pesticides, pose a potential risk as triggers of undesired immunoreactions or pathogenesis. Besides that, the purity of the isolates reflects on the efficacy and reproducibility of the electrospinning process as well as on the electrospun material endpoint functionality [17]. Improvement of material stability by adding cross-linkers and co-polymers in quantity sometimes larger than the actual protein, increases the process complexity and prolongs its optimization. Another issue with plant proteins is their limited water solubility. Successful electrospinning with water or mild buffers was proven so far only for canola and soy proteins. Using food-grade or generally recognized-as-safe solvents for electrospun biomedical materials is of the utmost requirement since harsh organic solvents bring risks of toxicity and environmental hazards [68]. However, water is not a perfect protein solvent because many proteins are quite hydrophobic and tend to aggregate or poorly unfold. Due to its surface tension, using water can lead to non-continuous electrospinning processes and heterogenous fiber structures. Recently, hopes have been raised with employment of green eutectic solvent for zein electrospinning, which is a completely novel approach in electrospinning technology [119].

On the whole, plant-derived proteins are a promising platform for development of new electrospun materials relevant in biomedicine, with considerable contribution to agricultural sustainability. Bringing favorable outcome in both domains, biomedical products with plant protein fibers have great prospects of finally stepping into the market.

## Figures and Tables

**Table 3:** Electrospun wheat gluten and its potential biomedical application.

Protein	Adjuvant Polymer/Solvent	Electrospinning Type	Drug, Bioactive Compound and/or Condition Tested	Biomedical Effects and Suggested Application of the Electrospun Material	Reference
Wheat gluten	None/Buffer solution, SDS	Uniaxial	Using non-reducing solvents	Improved absorption of biofluids	[87]
	None/Acetic acid	Uniaxial	Glycerol monolaurate in WG	Antibacterial in vitro, Food packaging	[90]
	None/Acetic acid, Alcohols, Acetone	Uniaxial	Urea in WG	Wound healing	[83]
	PVA/Acetic acid, Ethanol, 2-Propanol	Uniaxial	Azathioprine in WG/PVA blend	Drug release	[91]
	* C: None/ Hexafluoroisopropanol ** S: None/ Trifluoroacetic acid, Hexafluoroisopropanol	Coaxial	Ketoprofen (K); Monolyth Gliadin/K vs. Core–shell Gliadin/K-Gliadin	Drug release improved with coaxial system	[92]

* C—Core; ** S—Shell.

**Table 4:** Electrospun plant proteins and their potential biomedical application.

Protein	Adjuvant Polymer/Solvent	Electrospinning Type	Drug, Bioactive Compound and/or Condition Tested	Biomedical Effects and Suggested Application of the Electrospun Material	Reference
Pea protein	PVA/Alkaline and Acidic solutions	Uniaxial	Cinnamaldehyde in PP/PVA blend	Antibacterial food packaging	[95]
Canola protein	PEO/Water	Uniaxial	Clove essential oil	Antibacterial packaging	[105]
Taro protein	PEO, Chitosan/Acetic acid	Uniaxial	Crosslinking with glutaraldehyde and heat treatment	Antibacterial; Skin fibroblast growth supporting; Wound healing	[110]
Amaranth protein	Pullulan (P)/Formic acid	Uniaxial	Curcumin, Quercetin and Ferulic acid in AP/P blends	Drug release upon digestion in vitro	[115,116]

## Data Availability

Not applicable.

## Abbreviations

AP	amaranth protein
BP	bean protein
CP	canola protein
PCL	polycaprolactone
PEO	polyethylene oxide
PLA	polylactic acid
PP	pea protein
PtP	potato protein
PVA	polyvinyl alcohol
SF	silk fibroin
SFP	sunflower protein
SP	soy protein
TP	taro protein
WG	wheat gluten

## References

[1] Niva M., Vainio A. (2021). Towards more environmentally sustainable diets? Changes in the consumption of beef and plant- and insect-based protein products in consumer groups in Finland. Meat Sci..

[2] Karaś M., Jakubczyk A., Szymanowska U., Złotek U., Zielińska E. (2017). Digestion and bioavailability of bioactive phytochemicals. Int. J. Food Sci. Technol..

[3] Langyan S., Yadava P., Khan F.N., Dar Z.A., Singh R., Kumar A. (2022). Sustaining Protein Nutrition Through Plant-Based Foods. Front. Nutr..

[4] Gençdağ E., Görgüç A., Yılmaz F.M. (2021). Recent Advances in the Recovery Techniques of Plant-Based Proteins from Agro-Industrial By-Products. Food Rev. Int..

[5] Pojić M., Mišan A., Tiwari B. (2018). Eco-innovative technologies for extraction of proteins for human consumption from renewable protein sources of plant origin. Trends Food Sci. Technol..

[6] Kumar M., Tomar M., Potkule J., Verma R., Punia S., Mahapatra A., Belwal T., Dahuja A., Joshi S., Berwal M.K. (2021). Advances in the plant protein extraction: Mechanism and recommendations. Food Hydrocoll..

[7] Silva N.H.C.S., Vilela C., Marrucho I.M., Freire C.S.R., Pascoal Neto C., Silvestre A.J.D. (2014). Protein-based materials: From sources to innovative sustainable materials for biomedical applications. J. Mater. Chem. B.

[8] Reddy N., Yang Y. (2011). Potential of plant proteins for medical applications. Trends Biotechnol..

[9] Yıldız A., Kara A.A., Acartürk F. (2020). Peptide-protein based nanofibers in pharmaceutical and biomedical applications. Int. J. Biol. Macromol..

[10] Ghorbani M., Mahmoodzadeh F., Yavari Maroufi L., Nezhad-Mokhtari P. (2020). Electrospun tetracycline hydrochloride loaded zein/gum tragacanth/poly lactic acid nanofibers for biomedical application. Int. J. Biol. Macromol..

[11] Khabbaz B., Solouk A., Mirzadeh H. (2019). Polyvinyl alcohol/soy protein isolate nanofibrous patch for wound-healing applications. Prog. Biomater..

[12] Senthil Muthu Kumar T., Senthil Kumar K., Rajini N., Siengchin S., Ayrilmis N., Varada Rajulu A. (2019). A comprehensive review of electrospun nanofibers: Food and packaging perspective. Compos. Part B Eng..

[13] Barud H.S., De Sousa F.B. (2022). Electrospun Materials for Biomedical Applications. Pharmaceutics.

[14] Popov Pereira da Cunha M.D., Caracciolo P.C., Abraham G.A. (2021). Latest advances in electrospun plant-derived protein scaffolds for biomedical applications. Curr. Opin. Biomed. Eng..

[15] Uddin M.N., Jobaer M., Mahedi S.I., Ali A. (2022). Protein–based electrospun nanofibers: Electrospinning conditions, biomedical applications, prospects, and challenges. J. Text. Inst..

[16] İnce Yardamici A., Tarhan Ö. (2020). Electropsun Protein Nanofibers and Their Potential Food Applications. Mugla J. Sci. Technol..

[17] Aguilar-Vázquez G., Ortiz-Frade L., Figueroa-Cárdenas J.D., López-Rubio A., Mendoza S. (2020). Electrospinnability study of pea (Pisum sativum) and common bean (*Phaseolus vulgaris* L.) using the conformational and rheological behavior of their protein isolates. Polym. Test..

[18] Mendes A.C., Stephansen K., Chronakis I.S. (2017). Electrospinning of food proteins and polysaccharides. Food Hydrocoll..

[19] Akhmetova A., Heinz A. (2020). Electrospinning Proteins for Wound Healing Purposes: Opportunities and Challenges. Pharmaceutics.

[20] Aman Mohammadi M., Rostami M.R., Raeisi M., Tabibi Azar M. (2018). Production of Electrospun Nanofibers from Food Proteins and Polysaccharides and Their Applications in Food and Drug Sciences. Jorjani Biomed. J..

[21] Nieuwland M., Geerdink P., Brier P., van den Eijnden P., Henket J.T.M.M., Langelaan M.L.P., Stroeks N., van Deventer H.C., Martin A.H. (2014). Reprint of “Food-grade electrospinning of proteins”. Innov. Food Sci. Emerg. Technol..

[22] Bucci R., Georgilis E., Bittner A.M., Gelmi M.L., Clerici F. (2021). Peptide-based electrospun fibers: Current status and emerging developments. Nanomaterials.

[23] Guan T., Zhang Z., Li X., Cui S., McClements D.J., Wu X., Chen L., Long J., Jiao A., Qiu C. (2022). Preparation, Characteristics, and Advantages of Plant Protein-Based Bioactive Molecule Delivery Systems. Foods.

[24] Babitha S., Rachita L., Karthikeyan K., Shoba E., Janani I., Poornima B., Purna Sai K. (2017). Electrospun protein nanofibers in healthcare: A review. Int. J. Pharm..

[25] Zhu X., Kim K. (2022). Electrospun polyacrylonitrile fibrous membrane for dust removal. Front. Mater..

[26] Torres-Giner S., Gimenez E., Lagaron J.M. (2008). Characterization of the morphology and thermal properties of Zein Prolamine nanostructures obtained by electrospinning. Food Hydrocoll..

[27] Mosher C.Z., Brudnicki P.A.P., Gong Z., Childs H.R., Lee S.W., Antrobus R.M., Fang E.C., Schiros T.N., Lu H.H. (2021). Green electrospinning for biomaterials and biofabrication. Biofabrication.

[28] Zdraveva E., Mijovic B. (2023). Frontier electrospun fibers for nanomedical applications. Biotechnology—Biosensors, Biomaterials and Tissue Engineering.

[29] Wang Y., Yu D.-G., Liu Y., Liu Y.-N. (2022). Progress of Electrospun Nanofibrous Carriers for Modifications to Drug Release Profiles. J. Funct. Biomater..

[30] Chen S.C., Huang X.B., Cai X.M., Lu J., Yuan J., Shen J. (2012). The influence of fiber diameter of electrospun poly(lactic acid) on drug delivery. Fibers Polym..

[31] Liu Y., Chen X., Gao Y., Yu D.-G., Liu P. (2022). Elaborate design of shell component for manipulating the sustained release behavior from core–shell nanofibres. J. Nanobiotechnol..

[32] Singh B., Shukla N., Kim J., Kim K., Park M.-H. (2021). Stimuli-Responsive Nanofibers Containing Gold Nanorods for On-Demand Drug Delivery Platforms. Pharmaceutics.

[33] Fredenberg S., Wahlgren M., Reslow M., Axelsson A. (2011). The mechanisms of drug release in poly(lactic-co-glycolic acid)-based drug delivery systems—A review. Int. J. Pharm..

[34] Reineccius G., Meng Y. (2023). Gelatin and other proteins for microencapsulation. Microencapsulation in the Food Industry.

[35] Yang J., Hu L., Cai T., Chen Q., Ma Q., Yang J., Meng C., Hong J. (2018). Purification and identification of two novel antioxidant peptides from perilla (*Perilla frutescens* L. Britton) seed protein hydrolysates. PLoS ONE.

[36] Tortorella S., Maturi M., Vetri Buratti V., Vozzolo G., Locatelli E., Sambri L., Comes Franchini M. (2021). Zein as a versatile biopolymer: Different shapes for different biomedical applications. RSC Adv..

[37] Yan X., Li M., Xu X., Liu X., Liu F. (2022). Zein-based nano-delivery systems for encapsulation and protection of hydrophobic bioactives: A review. Front. Nutr..

[38] Pérez-Guzmán C.J., Castro-Muñoz R. (2020). A Review of Zein as a Potential Biopolymer for Tissue Engineering and Nanotechnological Applications. Processes.

[39] Turasan H., Cakmak M., Kokini J. (2022). A disposable ultrasensitive surface enhanced Raman spectroscopy biosensor platform fabricated from biodegradable zein nanofibers. J. Appl. Polym. Sci..

[40] Soares A.C., Soares J.C., dos Santos D.M., Migliorini F.L., Popolin-Neto M., dos Santos Cinelli Pinto D., Carvalho W.A., Brandão H.M., Paulovich F.V., Correa D.S. (2023). Nanoarchitectonic E-Tongue of Electrospun Zein/Curcumin Carbon Dots for Detecting *Staphylococcus aureus* in Milk. ACS Omega.

[41] Aghaei Z., Ghorani B., Emadzadeh B., Kadkhodaee R., Tucker N. (2020). Protein-based halochromic electrospun nanosensor for monitoring trout fish freshness. Food Control.

[42] Paliwal R., Palakurthi S. (2014). Zein in controlled drug delivery and tissue engineering. J. Control. Release.

[43] Mitra S., Mateti T., Ramakrishna S., Laha A. (2022). A Review on Curcumin-Loaded Electrospun Nanofibers and their Application in Modern Medicine. JOM.

[44] Akrami-Hasan-Kohal M., Tayebi L., Ghorbani M. (2020). Curcumin-loaded naturally-based nanofibers as active wound dressing mats: Morphology, drug release, cell proliferation, and cell adhesion studies. New J. Chem..

[45] Jiang W., Zhao P., Song W., Wang M., Yu D.-G. (2022). Electrospun Zein/Polyoxyethylene Core-Sheath Ultrathin Fibers and Their Antibacterial Food Packaging Applications. Biomolecules.

[46] Martin A., Cai J., Schaedel A.-L., van der Plas M., Malmsten M., Rades T., Heinz A. (2022). Zein-polycaprolactone core–shell nanofibers for wound healing. Int. J. Pharm..

[47] Asadi H., Ghaee A., Nourmohammadi J., Mashak A. (2020). Electrospun zein/graphene oxide nanosheet composite nanofibers with controlled drug release as antibacterial wound dressing. Int. J. Polym. Mater. Polym. Biomater..

[48] Kimna C., Tamburaci S., Tihminlioglu F. (2019). Novel zein-based multilayer wound dressing membranes with controlled release of gentamicin. J. Biomed. Mater. Res. Part B Appl. Biomater..

[49] Surendranath M., Ramesan R.M., Nair P., Parameswaran R. (2023). Electrospun Mucoadhesive Zein/PVP Fibroporous Membrane for Transepithelial Delivery of Propranolol Hydrochloride. Mol. Pharm..

[50] Ghorbani M., Nezhad-Mokhtari P., Ramazani S. (2020). Aloe vera-loaded nanofibrous scaffold based on Zein/Polycaprolactone/Collagen for wound healing. Int. J. Biol. Macromol..

[51] Barakat H.S., Freag M.S., Gaber S.M., Al Oufy A., Abdallah O.Y. (2023). Development of Verapamil Hydrochloride-loaded Biopolymer-based Composite Electrospun Nanofibrous Mats: In Vivo Evaluation of Enhanced Burn Wound Healing without Scar Formation. Drug Des. Devel. Ther..

[52] Qin M., Mou X., Dong W., Liu J., Liu H., Dai Z., Huang X., Wang N., Yan X. (2020). In Situ Electrospinning Wound Healing Films Composed of Zein and Clove Essential Oil. Macromol. Mater. Eng..

[53] Deng L., Li Y., Zhang H. (2020). In vitro and in vivo assessment of glucose cross-linked gelatin/zein nanofibrous scaffolds for cranial bone defects regeneration. J. Biomed. Mater. Res. Part B Appl. Biomater..

[54] Zhao H., Ma Y., Sun D., Ma W., Yao J., Zhang M. (2019). Preparation and Characterization of Coaxial Electrospinning rhBMP2-Loaded Nanofiber Membranes. J. Nanomater..

[55] dos Santos D.M., de Annunzio S.R., Carmello J.C., Pavarina A.C., Fontana C.R., Correa D.S. (2022). Combining Coaxial Electrospinning and 3D Printing: Design of Biodegradable Bilayered Membranes with Dual Drug Delivery Capability for Periodontitis Treatment. ACS Appl. Bio Mater..

[56] Pedram Rad Z., Mokhtari J., Abbasi M. (2018). Fabrication and characterization of PCL/zein/gum arabic electrospun nanocomposite scaffold for skin tissue engineering. Mater. Sci. Eng. C.

[57] Qi G., Venkateshan K., Mo X., Zhang L., Sun X.S. (2011). Physicochemical Properties of Soy Protein: Effects of Subunit Composition. J. Agric. Food Chem..

[58] Tang C.-H. (2019). Nanostructured soy proteins: Fabrication and applications as delivery systems for bioactives (a review). Food Hydrocoll..

[59] Cho D., Netravali A.N., Joo Y.L. (2012). Mechanical properties and biodegradability of electrospun soy protein Isolate/PVA hybrid nanofibers. Polym. Degrad. Stab..

[60] Sett S., Lee M.W., Weith M., Pourdeyhimi B., Yarin A.L. (2015). Biodegradable and biocompatible soy protein/polymer/adhesive sticky nano-textured interfacial membranes for prevention of esca fungi invasion into pruning cuts and wounds of vines. J. Mater. Chem. B.

[61] Thirugnanaselvam M., Gobi N., Arun Karthick S. (2013). SPI/PEO blended electrospun martrix for wound healing. Fibers Polym..

[62] Vega-Lugo A.-C., Lim L.-T. (2009). Controlled release of allyl isothiocyanate using soy protein and poly(lactic acid) electrospun fibers. Food Res. Int..

[63] Stie M.B., Kalouta K., da Cunha C.F.B., Feroze H.M., Vetri V., Foderà V. (2022). Sustainable strategies for waterborne electrospinning of biocompatible nanofibers based on soy protein isolate. Sustain. Mater. Technol..

[64] Kutzli I., Gibis M., Baier S.K., Weiss J. (2019). Electrospinning of whey and soy protein mixed with maltodextrin—Influence of protein type and ratio on the production and morphology of fibers. Food Hydrocoll..

[65] da Silva F.T., da Cunha K.F., Fonseca L.M., Antunes M.D., El Halal S.L.M., Fiorentini Â.M., Zavareze E.d.R., Dias A.R.G. (2018). Action of ginger essential oil (*Zingiber officinale*) encapsulated in proteins ultrafine fibers on the antimicrobial control in situ. Int. J. Biol. Macromol..

[66] Raeisi M., Mohammadi M.A., Bagheri V., Ramezani S., Ghorbani M., Tabibiazar M., Coban O.E., Khoshbakht R., Marashi S.M.H., Noori S.M.A. (2023). Fabrication of Electrospun Nanofibres of Soy Protein Isolate/Polyvinyl Alcohol Embedded with Cinnamon Zeylanicum and Zataria Multiflora Essential Oils and their Antibacterial Effect. Biointerface Res. Appl. Chem..

[67] Kim D.Y., Chaudhry M.A., Kennard M.L., Jardon M.A., Braasch K., Dionne B., Butler M., Piret J.M. (2013). Fed-batch CHO cell t-PA production and feed glutamine replacement to reduce ammonia production. Biotechnol. Prog..

[68] Zheng H., Yan G., Marquez S., Andler S., Dersjant-Li Y., de Mejia E.G. (2020). Molecular size and immunoreactivity of ethanol extracted soybean protein concentrate in comparison with other products. Process Biochem..

[69] Wongkanya R., Chuysinuan P., Pengsuk C., Techasakul S., Lirdprapamongkol K., Svasti J., Nooeaid P. (2017). Electrospinning of alginate/soy protein isolated nanofibers and their release characteristics for biomedical applications. J. Sci. Adv. Mater. Devices.

[70] Chuysinuan P., Pengsuk C., Lirdprapamongkol K., Techasakul S., Svasti J., Nooeaid P. (2019). Enhanced Structural Stability and Controlled Drug Release of Hydrophilic Antibiotic-Loaded Alginate/Soy Protein Isolate Core-Sheath Fibers for Tissue Engineering Applications. Fibers Polym..

[71] Doustdar F., Ramezani S., Ghorbani M., Mortazavi Moghadam F. (2022). Optimization and characterization of a novel tea tree oil-integrated poly (ε-caprolactone)/soy protein isolate electrospun mat as a wound care system. Int. J. Pharm..

[72] Gutschmidt D., Hazra R.S., Zhou X., Xu X., Sabzi M., Jiang L. (2021). Electrospun, sepiolite-loaded poly(vinyl alcohol)/soy protein isolate nanofibers: Preparation, characterization, and their drug release behavior. Int. J. Pharm..

[73] Ullah A., Sarwar M.N., Wang F., Kharaghani D., Sun L., Zhu C., Yoshiko Y., Mayakrishnan G., Lee J.S., Kim I.S. (2022). In vitro biocompatibility, antibacterial activity, and release behavior of halloysite nanotubes loaded with diclofenac sodium salt incorporated in electrospun soy protein isolate/hydroxyethyl cellulose nanofibers. Curr. Res. Biotechnol..

[74] Wei Z., Dai S., Huang J., Hu X., Ge C., Zhang X., Yang K., Shao P., Sun P., Xiang N. (2023). Soy Protein Amyloid Fibril Scaffold for Cultivated Meat Application. ACS Appl. Mater. Interfaces.

[75] Chatterjee C., Gleddie S., Xiao C.-W. (2018). Soybean Bioactive Peptides and Their Functional Properties. Nutrients.

[76] Ramji K., Shah R.N. (2014). Electrospun soy protein nanofiber scaffolds for tissue regeneration. J. Biomater. Appl..

[77] Xu H., Cai S., Sellers A., Yang Y. (2014). Intrinsically water-stable electrospun three-dimensional ultrafine fibrous soy protein scaffolds for soft tissue engineering using adipose derived mesenchymal stem cells. RSC Adv..

[78] Varshney N., Sahi A.K., Poddar S., Mahto S.K. (2020). Soy protein isolate supplemented silk fibroin nanofibers for skin tissue regeneration: Fabrication and characterization. Int. J. Biol. Macromol..

[79] Phelan M.A., Kruczek K., Wilson J.H., Brooks M.J., Drinnan C.T., Regent F., Gerstenhaber J.A., Swaroop A., Lelkes P.I., Li T. (2020). Soy Protein Nanofiber Scaffolds for Uniform Maturation of Human Induced Pluripotent Stem Cell-Derived Retinal Pigment Epithelium. Tissue Eng. Part C Methods.

[80] Zhang Q., Tong Z., Chen F., Wang X., Ren M., Zhao Y., Wu P., He X., Chen P., Chen Y. (2020). Aligned soy protein isolate-modified poly(L-lactic acid) nanofibrous conduits enhanced peripheral nerve regeneration. J. Neural Eng..

[81] Xu J., Li Y. (2023). Wheat gluten–based coatings and films: Preparation, properties, and applications. J. Food Sci..

[82] Woerdeman D.L., Ye P., Shenoy S., Parnas R.S., Wnek G.E., Trofimova O. (2005). Electrospun Fibers from Wheat Protein:  Investigation of the Interplay between Molecular Structure and the Fluid Dynamics of the Electrospinning Process. Biomacromolecules.

[83] Castro-Enríquez D., Rodríguez-Félix F., Ramírez-Wong B., Torres-Chávez P., Castillo-Ortega M., Rodríguez-Félix D., Armenta-Villegas L., Ledesma-Osuna A. (2012). Preparation, Characterization and Release of Urea from Wheat Gluten Electrospun Membranes. Materials.

[84] Dhandayuthapani B., Mallampati R., Sriramulu D., Dsouza R.F., Valiyaveettil S. (2014). PVA/Gluten Hybrid Nanofibers for Removal of Nanoparticles from Water. ACS Sustain. Chem. Eng..

[85] Lubasova D., Mullerova J., Netravali A.N. (2015). Water-resistant plant protein—Based nanofiber membranes. J. Appl. Polym. Sci..

[86] Dong J., Asandei A.D., Parnas R.S. (2010). Aqueous electrospinning of wheat gluten fibers with thiolated additives. Polymer.

[87] Muneer F., Hedenqvist M.S., Hall S., Kuktaite R. (2022). Innovative Green Way to Design Biobased Electrospun Fibers from Wheat Gluten and These Fibers’ Potential as Absorbents of Biofluids. ACS Environ. Au.

[88] Soares R.M.D., Patzer V.L., Dersch R., Wendorff J., da Silveira N.P., Pranke P. (2011). A novel globular protein electrospun fiber mat with the addition of polysilsesquioxane. Int. J. Biol. Macromol..

[89] Zhang Y., Deng L., Zhong H., Pan J., Li Y., Zhang H. (2020). Superior water stability and antimicrobial activity of electrospun gluten nanofibrous films incorporated with glycerol monolaurate. Food Hydrocoll..

[90] Zhang Y., Deng L., Zhong H., Zou Y., Qin Z., Li Y., Zhang H. (2022). Impact of glycation on physical properties of composite gluten/zein nanofibrous films fabricated by blending electrospinning. Food Chem..

[91] Aziz S., Hosseinzadeh L., Arkan E., Azandaryani A.H. (2019). Preparation of electrospun nanofibers based on wheat gluten containing azathioprine for biomedical application. Int. J. Polym. Mater. Polym. Biomater..

[92] Liu X., Shao W., Luo M., Bian J., Yu D.-G. (2018). Electrospun Blank Nanocoating for Improved Sustained Release Profiles from Medicated Gliadin Nanofibers. Nanomaterials.

[93] Ismail B.P., Senaratne-Lenagala L., Stube A., Brackenridge A. (2020). Protein demand: Review of plant and animal proteins used in alternative protein product development and production. Anim. Front..

[94] Lu Z.X., He J.F., Zhang Y.C., Bing D.J. (2020). Composition, physicochemical properties of pea protein and its application in functional foods. Crit. Rev. Food Sci. Nutr..

[95] Maftoonazad N., Shahamirian M., John D., Ramaswamy H. (2019). Development and evaluation of antibacterial electrospun pea protein isolate-polyvinyl alcohol nanocomposite mats incorporated with cinnamaldehyde. Mater. Sci. Eng. C.

[96] Jia X.W., Qin Z.Y., Xu J.X., Kong B.H., Liu Q., Wang H. (2020). Preparation and characterization of pea protein isolate-pullulan blend electrospun nanofiber films. Int. J. Biol. Macromol..

[97] Kutzli I., Griener D., Gibis M., Schmid C., Dawid C., Baier S.K., Hofmann T., Weiss J. (2020). Influence of Maillard reaction conditions on the formation and solubility of pea protein isolate-maltodextrin conjugates in electrospun fibers. Food Hydrocoll..

[98] OuYang Q., Duan X., Li L., Tao N. (2019). Cinnamaldehyde Exerts Its Antifungal Activity by Disrupting the Cell Wall Integrity of Geotrichum citri-aurantii. Front. Microbiol..

[99] Kennedy K., Cal R., Casey R., Lopez C., Adelfio A., Molloy B., Wall A.M., Holton T.A., Khaldi N. (2020). The anti-ageing effects of a natural peptide discovered by artificial intelligence. Int. J. Cosmet. Sci..

[100] Ramírez-Jiménez A.K., Reynoso-Camacho R., Mendoza-Díaz S., Loarca-Piña G. (2014). Functional and technological potential of dehydrated *Phaseolus vulgaris* L. flours. Food Chem..

[101] Rahmati N.F., Koocheki A., Varidi M., Kadkhodaee R. (2018). Introducing Speckled sugar bean (*Phaseolus vulgaris*) protein isolates as a new source of emulsifying agent. Food Hydrocoll..

[102] Yao F., Gao Y., Chen F., Du Y. (2022). Preparation and properties of electrospun peanut protein isolate/poly-l-lactic acid nanofibers. LWT.

[103] Campbell L., Rempel C., Wanasundara J. (2016). Canola/Rapeseed Protein: Future Opportunities and Directions—Workshop Proceedings of IRC 2015. Plants.

[104] Wanasundara J.P.D. (2011). Proteins of *Brassicaceae* Oilseeds and their Potential as a Plant Protein Source. Crit. Rev. Food Sci. Nutr..

[105] Zhao Y.-M., Li Y., Ma H., He R. (2023). Effects of ultrasonic-assisted pH shift treatment on physicochemical properties of electrospinning nanofibers made from rapeseed protein isolates. Ultrason. Sonochem..

[106] Pramod K., Ansari S.H., Ali J. (2010). Eugenol: A Natural Compound with Versatile Pharmacological Actions. Nat. Prod. Commun..

[107] Shanesazzadeh E., Kadivar M., Fathi M. (2018). Production and characterization of hydrophilic and hydrophobic sunflower protein isolate nanofibers by electrospinning method. Int. J. Biol. Macromol..

[108] Waglay A., Karboune S. (2016). Potato Proteins. Advances in Potato Chemistry and Technology.

[109] SHEWRY P.R. (2003). Tuber Storage Proteins. Ann. Bot..

[110] Wardhani R.A.K., Asri L.A.T.W., Rachmawati H., Khairurrijal K., Purwasasmita B.S. (2020). Physical–Chemical Crosslinked Electrospun *Colocasia esculenta* Tuber Protein–Chitosan–Poly(Ethylene Oxide) Nanofibers with Antibacterial Activity and Cytocompatibility. Int. J. Nanomedicine.

[111] Gibis M., Pribek F., Weiss J. (2022). Effects of Electrospun Potato Protein–Maltodextrin Mixtures and Thermal Glycation on Trypsin Inhibitor Activity. Foods.

[112] Gibis M., Pribek F., Kutzli I., Weiss J. (2021). Influence of the Protein Content on Fiber Morphology and Heat Treatment of Electrospun Potato Protein–Maltodextrin Fibers. Appl. Sci..

[113] Janssen F., Pauly A., Rombouts I., Jansens K.J.A., Deleu L.J., Delcour J.A. (2017). Proteins of Amaranth (*Amaranthus* spp.), Buckwheat (*Fagopyrum* spp.), and Quinoa (*Chenopodium* spp.): A Food Science and Technology Perspective. Compr. Rev. Food Sci. Food Saf..

[114] Aceituno-Medina M., Mendoza S., Lagaron J.M., López-Rubio A. (2013). Development and characterization of food-grade electrospun fibers from amaranth protein and pullulan blends. Food Res. Int..

[115] Blanco-Padilla A., López-Rubio A., Loarca-Piña G., Gómez-Mascaraque L.G., Mendoza S. (2015). Characterization, release and antioxidant activity of curcumin-loaded amaranth-pullulan electrospun fibers. LWT—Food Sci. Technol..

[116] Aceituno-Medina M., Mendoza S., Rodríguez B.A., Lagaron J.M., López-Rubio A. (2015). Improved antioxidant capacity of quercetin and ferulic acid during in-vitro digestion through encapsulation within food-grade electrospun fibers. J. Funct. Foods.

[117] Soto K.M., Hernández-Iturriaga M., Loarca-Piña G., Luna-Bárcenas G., Gómez-Aldapa C.A., Mendoza S. (2016). Stable nisin food-grade electrospun fibers. J. Food Sci. Technol..

[118] Zhang H., Jin C., Lv S., Ren F., Wang J. (2023). Study on electrospinning of wheat gluten: A review. Food Res. Int..

[119] Khatri M., Khatri Z., El-Ghazali S., Hussain N., Qureshi U.A., Kobayashi S., Ahmed F., Kim I.S. (2020). Zein nanofibers via deep eutectic solvent electrospinning: Tunable morphology with super hydrophilic properties. Sci. Rep..

